# Older Versus Younger Men Who Have Sex with Men: Awareness of and Potential Barriers to the Use of Pre-Exposure Prophylaxis (PrEP) Medication to Prevent the Transmission of HIV

**DOI:** 10.33696/aids.2.006

**Published:** 2020

**Authors:** Hugh Klein, Thomas Alex Washington

**Affiliations:** 1 Kensington Research Institute, Silver Spring, Maryland, United States; 2 California State University, Long Beach, California, United States

**Keywords:** Older MSM, Younger MSM, Pre-exposure prophylaxis (PrEP), Perceived stigma, Perceived obstacles

## Abstract

**Purpose:**

This paper compares younger (aged 18–39; n=197) and older (ages 50+; n=53) MSM to determine their familiarity with PrEP, willingness to learn more about PrEP, perceptions of stigma relating to PrEP use, and perceptions of barriers to PrEP adoption.

**Methods:**

A purposive sample of diverse MSM completed 15-minute questionnaires. Younger and older MSM were compared using Student’s t-tests and odds ratios for bivariate analyses, and multivariate logistic regression and multiple regressions for analyses controlling for key demographic characteristics.

**Results:**

Compared to younger MSM, older MSM were more aware of PrEP, more likely to know another PrEP user, less interested in learning more about PrEP, and more averse to using existing resources to learn more about PrEP. Older men perceived less stigma relating to PrEP and fewer obstacles needing to be overcome in order to give serious consideration to PrEP adoption. These differences remained when race, educational attainment, sexual orientation, and HIV serostatus were controlled.

**Conclusions:**

There is a “good news/bad news” situation with respect to older MSM and PrEP. They were more aware of PrEP, less likely to associate stigma with PrEP use or PrEP users, and anticipated fewer barriers to PrEP adoption. They were also less interested than their younger counterparts in learning more about PrEP and expressed less comfort using existing sources of information to learn more about PrEP. Age-appropriate PrEP educational campaigns are advisable in order to reach older MSM and encourage more of them to consider PrEP adoption.

## Introduction

Men who have sex with other men (MSM) comprise the single largest group of individuals contracting HIV in the United States, accounting for more than one-half of all new HIV diagnoses [1]. These persons have been the single most affected of all population groups throughout all of the years of the HIV/AIDS epidemic [1–3]. In recent years, coinciding with this, there has been a trend for older adults (defined here as persons aged 50 or older) to be at increasing risk for contracting HIV. Between 2002 and 2017, the proportion of all newly-diagnosed cases of HIV among people aged 50 or older rose slowly but steadily from 15.9% (2002) to 16.4% (2007) to 16.9% (2012) to 17.1% (2017) [1–3]. Now more than ever before, the data shows, older MSM are at risk for contracting HIV.

Researchers have documented that substantial proportions of older MSM are sexually active [4–6], with many of these persons reporting inconsistent condom use [6–8] or involvement in multiple HIV risk behaviors [4,5]. Additionally, illegal drug use, which has been linked in numerous studies to greater involvement in risky sex, has been found to be fairly prevalent among older MSM [5,7–9].

Despite these findings, comparatively little has been written about why older MSM engage in these higher-risk practices and, contemporarily, what role, if any pre-exposure prophylaxis (PrEP) plays in their ongoing efforts to try to avoid contracting HIV. (For readers who may not know much about PrEP, it is a medication, typically daily taken in pill form, to reduce the likelihood of contracting HIV. When used properly, PrEP reduces the chance of contracting HIV by anywhere from 86–93% [1,10,11]). Indeed, the present authors have been unable to identify any publishes studies examining PrEP use versus nonuse among older MSM. Given the fact that the United States’ Federal government agencies have, in recent years, placed PrEP at the forefront of ongoing efforts to prevent the spread of HIV, this leaves a noteworthy gap in knowledge. Data have not been provided about what proportion of older MSM who engage in risky behaviors currently use PrEP. Moreover, data have not been provided to address the issue of what percentage of older MSM is even aware of PrEP. (Numerous studies, for example, have shown that PrEP awareness is low among other populations of MSM [12–14]; but these studies were not based solely or even largely on older MSM populations.) Among those who are aware of the medication, information is lacking regarding the factors that influence their decisions to speak with their physicians about the possible adoption of PrEP and, ultimately, their decisions to give the medication a try versus not doing so.

The present study represents an effort to begin bridging this gap in knowledge. Here, the present authors rely upon a purposive sample of MSM, divided strategically into younger men (those aged 18–39) and older men (those aged 50 and older), and address the following questions: (1) Are there differences between older and younger MSM with respect to PrEP awareness? (2) Are there differences between older and younger MSM with regard to previous exposure to people who have used PrEP? (3) Do younger and older MSM differ with respect to their level of interest in learning more about PrEP? (4) Are there age-related differences in willingness to avail oneself of existing resources for additional information about PrEP? (5) Do older and younger MSM differ in their perceptions of stigma relating to the potential use of PrEP? (6) Are there differences between older and younger MSM with respect to their perceptions of obstacles needing to be overcome in order to give PrEP medication more serious consideration?

## Methods

### Sample

A purposive sampling approach was used to derive the final research population for this study. By choosing this methodological approach, the researchers’ principal goal was to assemble as diverse a sample of MSM as possible. In this manner, the present authors are able to examine differences among different subgroups of MSM–for example, Caucasians versus African Americans versus Latinos, or younger men versus older men–by virtue of each subgroup’s representation in the final sample. Typically, it is this quality of purposive sampling that is cited as one of its greatest strengths and most advantageous uses, along with the fact that, when implemented properly, it yields results that are comparable to more-scientifically-sound methodological approaches even though purposive sampling itself is a nonrandom sampling approach.

For this study, which was conducted between November 2017 and June 2018, 273 men were recruited via four distinct yet strategically-chosen approaches: The first entailed approaching men participating in a few different social/activities/support groups for MSM and asking them to take part in the study. The second involved a research assistant asking men attending a local Gay Pride event if they would be willing to take part in the study. The third entailed posting a profile on one particular dating/sex site targeting MSM of all ages and racial/ethnic groups, logging onto that website, and sending a generic “hello” type of message to initiate a casual conversation with anyone who visited the profile while the researcher was logged on. The fourth approach consisted of asking participants enrolled into the study via any of the first three methods to speak with friends and acquaintances of theirs, to see if they could get some of them to take part in the study. The research protocol was approved by the institutional review board at California State University–Long Beach.

### Procedures

Would-be participants were given the opportunity to ask questions about the study, and then they were asked if they remained interested in participating. For those men who were enrolled into the study via one of the face-to-face methods of recruitment, verbal informed consent was provided before the questionnaire was administered. For men who were enrolled into the study via one of the electronic recruitment methods, acknowledgment of their willingness to participate in the research via email was obtained before a copy of the questionnaire was sent to them for completion. The questionnaire took approximately 15 minutes to complete and no compensation was offered. The survey instrument consisted of a few brief sections. Basic demographic information was collected in one section. In another, familiarity with PrEP and other PrEP users was examined, as was their level of interest in obtaining additional information about PrEP. Participants were asked about their likelihood of availing themselves of various types of sources for obtaining additional information about PrEP. In the final section of the questionnaire, items comprising the PrEP Obstacles Scale (described below) and the PrEP Stigma Scale (described below) were included. Participants who were given the opportunity to answer the questionnaire in the presence of the research assistant completed their survey manually and simply handed their completed answer sheet to that individual when they were done. Those who came to the project via contact referrals or from the dating/sex website were asked to email their completed answer sheet (or a photograph or scanned copy of their completed answer sheet) to a project-sponsored email account. Participants were told that their identity would remain private, and that their answers and email addresses (used for returning completed answer sheets to the research team) would be kept confidential and would not be shared with anyone else. When they had submitted their completed answer sheet to the appropriate member of the research team, men were thanked for their time and participation, and then asked to contact other potentially-eligible and potentially-interested MSM they knew to help expand the sample. Respondents were not asked for their name, telephone number, email address, or any other personally-identifying information, so that their participation could be as private and confidential as possible.

### Measures

Demographic information collected in the questionnaire consisted of age (continuous), race/ethnicity (Caucasian, African American, Latino, Asian/Pacific Islander, Native American, or biracial/multiracial), relationship status (single, engaged or seriously involved with someone, married or involved in a long-term relationship), educational attainment (ordinal), sexual orientation (self-reported as gay, bisexual, or heterosexual), and HIV serostatus (self-reported as HIV-negative, HIV-positive, or serostatus unknown).

Knowledge and Understanding of PrEP consisted of items asking whether or not men had ever heard of PrEP prior to participating in this study (yes/no), whether or not they personally knew any PrEP users (yes/no), and how accurate their understanding of PrEP was prior to participating in the study once they were given a project-provided explanation of what PrEP is (five-point ordinal measure, ranging from “not at all accurate” to “very accurate”).

Interest in Learning More about PrEP was assessed by asking men how interested they were right now in learning more about PrEP (five-point ordinal measure, ranging from “not at all interested” to “very interested”). Then, separate questions were asked about how likely men thought they were to seek additional information about PrEP sometime during the next three months by (1) speaking with their friends, (2) asking their healthcare provider or personal physician, (3) visiting websites or watching podcasts, (4) going to the local health department, (5) reading postings on social media sites, or (6) contacting people on sex or dating websites or phone apps. Responses to these five-point ordinal items ranged from “not at all likely” to “very likely.” A PrEP Resources Scale measure was constructed from these six items, with higher scores indicating a greater overall willingness to avail oneself of various PrEP information sources. The scale was found to be reliable (Cronbach’s alpha=0.88).

Perceived Stigma was assessed via the 22-item PrEP Stigma Scale [15]. All items were scored on a five-point Likert scale with responses ranging from “strongly agree” to “strongly disagree.” The underlying intent (and the focus) of these items was to explore potential sources of stigma that men associated with PrEP use, in the event that they would ever decide to consider adopting it for themselves. Among others, these stigmata included a perception that using PrEP meant that one was promiscuous, concern that one’s sex partner(s) would think that one was engaging in risky sex with other men if that person were found out to be a PrEP user, concern that one’s friends and/or family members would think less highly of him if they were to discover that he used PrEP, fear of being ostracized or avoided by friends if they were to learn that the man used PrEP, concern about being treated differently in health care settings and/or during doctors’ visits if the staff found out that the person used PrEP, and fear of people sharing information about one’s PrEP use with other persons without obtaining permission to do so beforehand. The scale was found to be highly reliable, both for the sample as a whole and for all subgroups based on race, age, relationship status, educational attainment, sexual orientation, and HIV serostatus (all Cronbach’s alpha values were 0.88 or greater).

Perceived Obstacles Needing to Be Overcome was assessed via the 20-item PrEP Obstacles Scale [16]. All items were scored on a five-point Likert scale with responses ranging from “strongly agree” to “strongly disagree.” The underlying intent and focus of these items was to explore potential obstacles that men might perceive as needing to be overcome in the event that they would ever decide to consider adopting PrEP (or give serious consideration to adopting it) for themselves. Among others, these obstacles included not knowing enough about PrEP to allow the individual to make an informed decision about using/not using it, concerns about the affordability and accessibility of PrEP medication, discomfort about discussing PrEP with one’s personal physician or sex partners (separate items), and concerns about possible side-effects or efficacy of the medication (separate items). The scale was found to be highly reliable, both for the sample as a whole and for all subgroups based on race, age, relationship status, educational attainment, sexual orientation, and HIV serostatus (all Cronbach’s alpha values were 0.84 or greater).

### Analysis

The Statistical Analysis Software (SAS), version 9.3, was used to perform all analytical functions. Study participants were divided into two age groups for analytical purposes– namely, those aged 18–39 (i.e., younger men) and those aged 50 or older (i.e., older men). Men aged 40–49 were excluded from this paper’s analyses so as to base the findings on the most meaningful intergroup differences possible (For example, had those excluded men been included in these analyses, people who were only one year apart in age–39 year olds and 40 year olds–would have been classified differently for analytical purposes. That, the present authors believe, would have created false comparison groups.).

To foster easy-to-understand intergroup comparisons in the statistical analyses, all of the demographic variables were recorded into dichotomous measures (e.g., single versus “involved,” HIV-positive versus HIV-negative, gay versus other-than-gay MSM, and so forth). Whenever the outcome measure was dichotomous (e.g., knowing versus not knowing about PrEP, knowing versus not knowing any PrEP users), odds ratios (OR) were used as the primary analytical tool, with 95% confidence intervals (*CI_95_*) reported for each point estimate. For the scales measuring participants’ willingness to avail themselves of potential information sources about PrEP, the PrEP Stigma Scale, and the PrEP Obstacles Scale, Student’s t tests were performed.

Comparisons of the demographic characteristics of the younger and the older men comprising the sample yielded several statistically-significant intergroup differences (see Sample subsection, below, and Table 1). This was true for race, educational attainment, sexual orientation, and HIV serostatus (but not relationship status). Consequently, these measures were used as control variables in the final step in the analysis, so as to illuminate whether age differences in the findings were sustained when the impact of known influential measures was taken into account statistically. This entailed the use of multivariate logistic regression for dichotomous outcome measures (e.g., knowing versus not knowing any PrEP users, had versus had not heard about PrEP previously) and multiple regression for continuous measures (e.g., overall stigma associated with PrEP, perceived obstacles needing to be overcome). Results are reported as statistically significant whenever p<0.05.

## Results

### Sample

The sample consists of 250 men, 197 of whom were aged 18–39 (younger) and 53 of whom were aged 50 or older (older). Table 1 presents details about the demographic composition of the sample as a whole and the age-based subgroups being compared in this paper. Slightly more than one-third of the participants were Caucasian (37.0%), with African Americans (27.1%) and Latinos (18.3%) comprising the two next-largest groups. The remaining 17.6% of the sample was comprised by Asians and Pacific Islanders (8.8%), Native Americans or Native Alaskans (1.5%), and men who self-identified as biracial or multiracial (7.3%). Older men were significantly more likely to be Caucasian than younger men were (p<.0001). Most of the men self-identified as gay (69.6%) but there was excellent representation as well from bisexual men (16.1%) and MSM who self-identified as heterosexual (14.3%). Older men were significantly more likely than younger men to consider themselves to be gay (p<0.02) whereas younger men were more likely to self-identify as bisexual (p<0.03). The large majority of the participants (80.6%) said that they were single and not involved in a steady relationship with anyone, compared to 8.8% who said that they were seriously dating or engaged to someone and 10.6% who said that they were married. Relationship status did not differ based on age group. The large majority of the respondents (82.1%) said that they were HIV-negative at the time of interview. Younger men were significantly more likely than older men to be HIV-negative, though (p<0.0001). Approximately 1 out of 9 men (11.0%) said that he had not completed high school or earned a G.E.D. This compares to 37.0% who had graduated from high school or earned a G.E.D., 34.1% who had some college education without the completion of a bachelor’s degree program, 8.4% who had completed college, and 9.5% who had earned either a master’s degree or a doctoral-level degree. Older men were significantly more likely to have at least a college degree than their younger counterparts (p<0.0001) (Table 1).

#### Part 1: Familiarity with PrEP:

Slightly more than one-quarter of the men (28.4%) had heard of PrEP prior to participating in this pilot study. Older men were considerably more likely than their younger counterparts to have been familiar with the medication (90.6% versus 11.7%; OR=72.63, *CI_95_*=26.23–201.11, p<0.0001). Age remained statistically significant even when the effects of race, educational attainment, sexual orientation, and HIV serostatus were taken into account (p<0.0001).

#### Part 2: Exposure to Actual PrEP Users:

Less than one-quarter of the study participants (22.4%) said that they personally knew at least one person who was currently or previously a PrEP user. Older men were much more likely than their younger counterparts to say that they personally knew at least one PrEP user (81.1% versus 6.6%; OR=60.86, CI_95_=25.02–148.02, p<0.0001). Age remained statistically significant even when the effects of race, educational attainment, sexual orientation, and HIV serostatus were taken into account (p<0.0001).

#### Part 3: Interest in Learning More about PrEP:

The desire to learn more about PrEP was moderately high in this sample. 6.4% of the men said that they were “not at all” interested in learning more about PrEP, compared to 6.0% who said that they were “not very” interested, 17.3% who were “somewhat” interested, 38.2% who were “fairly” interested, and 32.1% who were “very” interested. Younger respondents were significantly more likely to fall into the latter two groups (79.7% versus 34.6%; *OR*=7.41, *CI_95_*=3.80–14.47, p<0.0001) and, conversely, older respondents were significantly more likely to fall into one of the first two groups (42.3% versus 3.6%; *OR*=15.32, *CI_95_*=6.44–36.42, p<0.0001). Age remained a significant predictor even when the effects of the other control variables were taken into account (p<0.0001).

#### Part 4: Willingness to Use Various PrEP Information Sources:

Overall, willingness to use various information sources to learn more about PrEP was moderate in this sample (mean=2.02 on the 0–4 scale, SD=0.97). Compared to their older counterparts, younger men were willing to consider turning to a wider variety of sources of information about PrEP (2.21 versus 1.19; t=7.64, p<0.0001). Younger men were more willing than older men to say that they were likely to turn to friends (2.07 versus 1.42; t=3.39, p<0.0008), their personal physician (2.71 versus 1.30; t=8.37, p<0.0001), online resources such as podcasts or websites (2.99 versus 1.38; t=9.18, p<0.0001), the local health department (1.89 versus 1.06, t=5.17, p<0.0001), or social media websites (2.52 versus 1.08, t=7.50, p<0.0001), but not to sex or dating websites or cellphone apps (1.08 versus 0.89, t=1.23, n.s.) for information about PrEP. Age was a statistically significant contributor in the multivariate model that also examined the effects of race, educational attainment, sexual orientation, and HIV serostatus (p<0.0003).

#### Part 5: Perceived Stigma Associated with Using PrEP:

Overall, respondents perceived a moderate amount of stigma to be associated with the use of PrEP (mean=2.87 on a 1–5 scale, SD=0.57). Younger men had significantly higher perceived stigma scores than older men did (3.19 versus 1.66; t=17.14, p<0.0001). In the multivariate equation, age was the strongest contributor to the model when race, educational attainment, sexual orientation, and HIV serostatus were also considered (p<0.0001).

Among younger men, the most consequential stigma-related concerns were:

- Others would think that the person was having sex with many different partners if they were to find out that they were using PrEP (88.3%),
- Others would think that the person was engaging in strange types of sex if they were to discover that the person was using PrEP (88.3%),
- PrEP is intended for men who are unable to use condoms (81.6%),
- Their boyfriend/partner would think that they were having sex with other people if that person were to learn that the person was using PrEP (79.1%),
- Their health insurance premiums would increase if their insurer learned that they were using PrEP (68.0%), and
- PrEP is intended for men who consider themselves to be sexual “bottoms” (66.5%).

In clear contrast, the older men were much, much less apt to perceive stigma relating to the use of PrEP. Their top stigma-related concerns were:

- Their boyfriend/partner would think that they were having sex with other people if that person were to learn that the person was using PrEP (15.1%) and
- People would think that they were engaging in sex with many different partners if they were to learn that the person was using PrEP (13.2%).

#### Part 6: Perceived Obstacles to Using PrEP:

Study participants perceived there to be a fair number of obstacles needing to be overcome if they themselves were to give PrEP use greater consideration (mean=3.10 on a 1–5 scale, SD=0.50). Younger men perceived a greater number of obstacles precluding them from exploring PrEP use further when compared to their older counterparts (3.44 versus 1.83; t=20.68, p<0.0001). Age was the strongest contributor to the multivariate equation that also examined the effects of race, educational attainment, sexual orientation, and HIV serostatus (p<0.0001).

- Among younger men, the most commonly-cited perceived obstacles to exploring PrEP use further were:
- not knowing enough about PrEP in order to make an informed decision about using versus not using it (88.8%),
- not knowing enough about what PrEP does (85.3%),
- not knowing what might happen to one’s health if, in the future, one were to decide to cease using PrEP (84.3%),
- concerns about the newness of PrEP precluding scientists from knowing what the long-term consequences of use may be (84.2%),
- concerns about possible drug interactions with medications the person already takes (79.6%),
- lacking confidence in one’s ability to remember to take the PrEP medication daily, as required for efficacy (79.2%),
- disliking the idea of taking a medication when one is not suffering from any type of actual illness or disease (77.2%), and
- believing that there are easier ways to prevent HIV than by taking PrEP (66%).

Among older men, perceptions of obstacles needing to be overcome in order to give PrEP use more serious consideration were far, far lesser. Their greatest issues were:

- concerns about the newness of PrEP precluding scientists from knowing what the long-term consequences of use may be (13.2%),
- believing that there are easier ways to prevent HIV than by taking PrEP (11.5%), and
- believing that there are better ways to prevent HIV than by taking PrEP (11.5%).

## Discussion

### Limitations of the study

Before discussing the implications of this research, we would like to acknowledge two limitations of this study. First, the findings presented in this paper are based on a research sample that was not derived via random sampling. Instead, the data were collected via a purposive sampling approach that was designed to maximize diversity within the target population, so that analyses could be performed with different subpopulations of MSM fostering comparisons of men based on their age, race, educational attainment, and so forth. The adoption of the purposive sampling approach successfully accomplished this goal, while making it impossible for us to know the extent to which these findings may or may not be generalized to MSM in general. Second, although the number of older men in this study (*n*=53) was sufficient to facilitate meaningful statistical analysis, ideally, there would be more such men available to the researchers so as to foster greater confidence in the findings. How–or even if–the findings obtained might have been changed had there been, say, 100 or 200 respondents designated as “older men” is unknown.

## Conclusions

When it comes to the issues that seem to be at the forefront regarding why they are not using PrEP, the present study’s data suggest that younger men and older men are quite different. With respect to the older men and PrEP-related matters, it is a true good news/bad news situation.

On the “good news” side of the equation, older men were much more likely than their younger counterparts to be aware of PrEP and to have had exposure to at least one person whom they knew was or had been a PrEP user. Other analyses conducted by the present investigators [16,17] has revealed that personally knowing others who use PrEP is associated with fewer concerns about using PrEP, lower rates of perceived stigma regarding the potential use of PrEP, and fewer perceived barriers needing to be overcome in order for one to give serious consideration to PrEP adoption. Moreover, given other research findings suggesting that even today, approximately a full decade into the existence of PrEP as a tool in the fight against the spread of HIV, many, if not most, MSM are unaware of PrEP [13,14,18,19], it is encouraging that the older men in the present study were more aware than their younger counterparts to have heard about PrEP.

Also on the “good news” side of the equation, men aged 50 or older perceived there to be less stigma associated with PrEP medication and the use of PrEP than their younger counterparts did. Similarly, compared to their peers under the age of 40, older men anticipated fewer obstacles needing to be overcome in order for them to give PrEP use more-serious consideration. These more-positive perceptions of PrEP among the older men bode well for them with respect to potential PrEP adoption. Recently, interest has been growing in the area of PrEP-related stigma perceptions [20–22], with other researchers occasionally commenting on the stifling effects that such stigma perceptions have on MSM’s willingness to use PrEP [23,24]. Therefore, the finding that the present study’s older men scored far lower on the PrEP stigma measures is a positive outcome for these men. Just the same, some scholars have pointed out that much more needs to be learned about stigma perceptions as they relate to PrEP [21,25]; and the present study’s findings support that claim. One particularly interesting and salient finding regarding stigma perceptions among the older men in the present study is that one of their greatest concerns about PrEP is the perception that other persons may link its use to promiscuity. Eaton and colleagues [26] discussed this exact stigma perception and said that, in their research, it was associated strongly with a disinterest in using PrEP. Much more research is needed to understand precisely how specific PrEP-related stigma perceptions are related to specific behaviors.

On the “bad news” side of things, the present study revealed that, compared to their younger counterparts, older men were less interested in learning more about PrEP. This may be due to the fact that most of the older men (93.7%) said that their pre-participation understanding of PrEP was either “fairly accurate” or “very accurate,” compared with only 38.5% of their younger counterparts. Thus, they may not want to know more about PrEP because they feel as if they already know enough about it. Nevertheless, at least some level of disconnect appears to be happening here, because older adults having been comprising a steadily but increasingly-greater proportion of all new HIV diagnoses in the Unites States. Between 2002 and 2017, the proportion of all newly-diagnosed cases of HIV among people aged 50 or older has risen steadily from 15.9% (2002) to 16.4% (2007) to 16.9% (2012) to 17.1% (2017) [1–3]. This slow but steady rise in the HIV incidence rates among older adults, coupled with their lack of interest in learning more about PrEP, is cause for concern for this population. More needs to be done by way of developing and implementing age-specific/age-appropriate HIV intervention messages targeting older MSM. Other researchers have commented on the need for age-appropriate HIV messaging and education/prevention/intervention initiatives targeting older adults [27–29], and the present study’s findings are consistent with those researchers’ recommendations.

Also on the “bad news” side of things, compared to their younger peers, older men were more opposed to availing themselves of various resources that are available to learn more about PrEP. This portends potential problems, because when/if they decide that they want or need to learn more about PrEP or give more serious consideration to adopting the medication, they are likely to be uncomfortable with most of the available options for learning more about PrEP. Overall, compared to their younger counterparts, older men were less willing to consider turning to their friends, their personal physician, online resources such as podcasts or websites, a local health department, or social media sites for information about PrEP. If they ever decide that they want to find out more about PrEP or explore its viability for their personal use, where would they turn or feel comfortable turning for relevant, helpful information?

## Summary

Relying upon a purposive sample of diverse American MSM, the present paper examined the reasons why older MSM have not adopted PrEP in an effort to remain safe from HIV infection. Evidence supported a conceptualizing the findings as representing a “good news/bad news” scenario. Regarding the former, compared to their younger counterparts, older were much more likely to have heard about PrEP and to be acquainted personally with at least one current or previous PrEP user. Older men were less apt to express concerns about stigma pertaining to PrEP use and they perceived there to be fewer obstacles needing to be overcome in order to give PrEP use more serious consideration. Conversely, they were less willing than their younger counterparts to express an interest in learning more about PrEP and they were more averse to turning to most of the available resources (e.g., friends, personal physician, websites) for information about PrEP.

## Figures and Tables

**Table 1:** Demographic Composition of Total Pilot Study Sample and Subsamples of Younger and Older Men.

	Total Sample (*n*=273)	Younger Men (*n*=197)	Older Men (*n*=53)

**Race** ***			
Caucasian	37.0	19.8	90.6
African American	27.1	34.0	7.5
Latino	18.3	23.4	0.0
All Others (Including Multiracial)	8.8	22.8	1.9

**Relationship Status**			
Single / Uninvolved	80.6	83.3	75.5
Married / Involved	19.4	16.7	24.5

**HIV Serostatus** ***			
HIV-Negative	82.1	91.9	50.9
HIV-Positive or Unknown	18.9	8.1	49.1

**Educational Attainment** ***			
High School Graduate or Less	48.0	61.4	15.1
Some College	34.0	31.5	43.4
College Graduate	18.0	7.1	41.5

**Sexual Orientation** *			
Gay	69.6	66.0	83.0
Bisexual	16.1	17.8	5.6
Heterosexual	14.3	16.2	11.3

* p<0.05

** p<0.01

*** p<0.001
